# Ancient Peruvians

**Published:** 1884-01

**Authors:** 


### ANCIENT PERUVIANS.

The editor of *Zahntechnische Reform*, in a kindly notice of the discussion concerning the decay found in pre-historic teeth, calls our attention to the fact that the ancient Peruvians were not without a kind of civilization of their own. Quite true, but the question at issue is whether dental caries is due to the vices of a *modern* civilization; whether the type of oral diseases has been materially changed by altered habits of life, by the present methods of the preparation of food, by sanitary condition, and sedentary pursuits. We have no expert testimony that any species of what might be denominated dentistry was practiced among that people. Casual observers are liable to confound post-mortem decorations of the dead, the placing of golden ornaments in the mouth, the gilding of the teeth, etc., for prosthetic operations performed during life. The ancient Peruvians spent much labor in the preparation of their dead for burial, and the mummied remains are in a very good state of preservation to-day.

